# S100B is selectively expressed by gray matter protoplasmic astrocytes and myelinating oligodendrocytes in the developing CNS

**DOI:** 10.1186/s13041-021-00865-9

**Published:** 2021-10-06

**Authors:** Junqing Du, Min Yi, Fang Zhou, Wanjun He, Aifen Yang, Mengsheng Qiu, Hao Huang

**Affiliations:** Institute of Life Sciences, College of Life and Environmental Sciences, College of Basic Medical Science, Hangzhou Normal University, Hangzhou, 311121 China

**Keywords:** S100B, Expression, Astrocytes, Oligodendrocytes

## Abstract

Studies on the development of central nervous system (CNS) primarily rely on the use of specific molecular markers for different types of neural cells. S100B is widely being used as a specific marker for astrocytes in the CNS. However, the specificity of its expression in astrocyte lineage has not been systematically investigated and thus has remained a lingering issue. In this study, we provide several lines of molecular and genetic evidences that S100B is expressed in both protoplasmic astrocytes and myelinating oligodendrocytes. In the developing spinal cord, S100B is first expressed in the ventral neuroepithelial cells, and later in ALDH1L1+/GS+ astrocytes in the gray matter. Meanwhile, nearly all the S100B+ cells in the white matter are SOX10+/MYRF+ oligodendrocytes. Consistent with this observation, S100B expression is selectively lost in the white matter in *Olig2*-null mutants in which oligodendrocyte progenitor cells (OPCs) are not produced, and dramatically reduced in *Myrf*-conditional knockout mutants in which OPCs fail to differentiate. Similar expression patterns of S100B are observed in the developing forebrain. Based on these molecular and genetic studies, we conclude that S100B is not a specific marker for astrocyte lineage; instead, it marks protoplasmic astrocytes in the gray matter and differentiating oligodendrocytes.

## Introduction

The vertebrate central nervus system (CNS) tissues are composed of several types of cells, including neural progenitors, neurons, astrocytes, oligodendrocytes and microglia. During development, it is generally thought that neural progenitor cells first give rise to neurons, followed by astrocytes and oligodendrocytes [1]. This developmental process is coordinated by a large cohort of intracellular factors and extracellular signals. Decoding the developmental trajectories of various cell lineages often requires specific molecular markers that label distinct cell types or different developmental stages. To date, marker genes specific for astrocyte lineage are still very limited, and the paucity of stage-specific markers have hindered our research on the origin and molecular specification of astrocytes.

GFAP is a well-known molecular marker for astrocytes. However, GFAP is predominantly expressed in white matter fibrous astrocytes and is expressed in radial glial progenitor cells as well [2–4]. Besides GFAP, S100B (S100 protein, beta polypeptide, neural), has also been used as an astrocyte biomarker for several decades [5, 6]. S100B is a Ca^2+^-binding protein and forms a homodimer through S-bond [7–9]. The dimer formation is necessary for its neurotrophic function, as mutations in cysteine residues that disrupt the dimer formation can cause immunological responses of astrocytes and microglia [10, 11]. Additionally, S100B has been implicated in several neurological diseases including Alzheimer’s disease, Parkinson disease and neuropathic pain [12–15]. Transgenic mice overexpressing S100B display altered synaptic plasticity and impaired spatial learning and social behaviors [16, 17], whereas inactivation of S100B induces chronic astrogliosis, enhances synaptic plasticity and increases the incidents of epilepsy and blood–brain barrier permeability [18–21].

Despite its functional diversity in the CNS, a large body of literatures have reported that S100B is specifically expressed in cells of astrocyte lineage in the brain and spinal cord [6, 22, 23]. At present, it is being widely used as a specific molecular marker for astrocytes in both developing and adult CNS tissues. However, the specificity of S100B in astrocytes has not been analyzed in details and verified at the molecular and genetic levels. In fact, emerging evidence suggests that S100B might also be expressed in cells of oligodendrocyte lineage, or even in neurons [24–27]. For instance, it was reported that in S100B-EGFP transgenic mice, EGFP is co-expressed with oligodendrocyte markers OLIG2, NG2 and CNPase [24]. Hence, in this study, we systematically characterized the expression pattern of S100B in the developing CNS by double immunostaining with a battery of specific markers against astrocytes and cells of oligodendrocyte-lineage in wild-type and genetic mouse mutants. We conclude that S100B marks both differentiating oligodendrocytes and gray matter astrocytes in the CNS, and thus can not be used as a reliable astrocyte marker.

## Results

### Expression of S100B in the developing spinal cord and forebrain

**Fig. 1:**
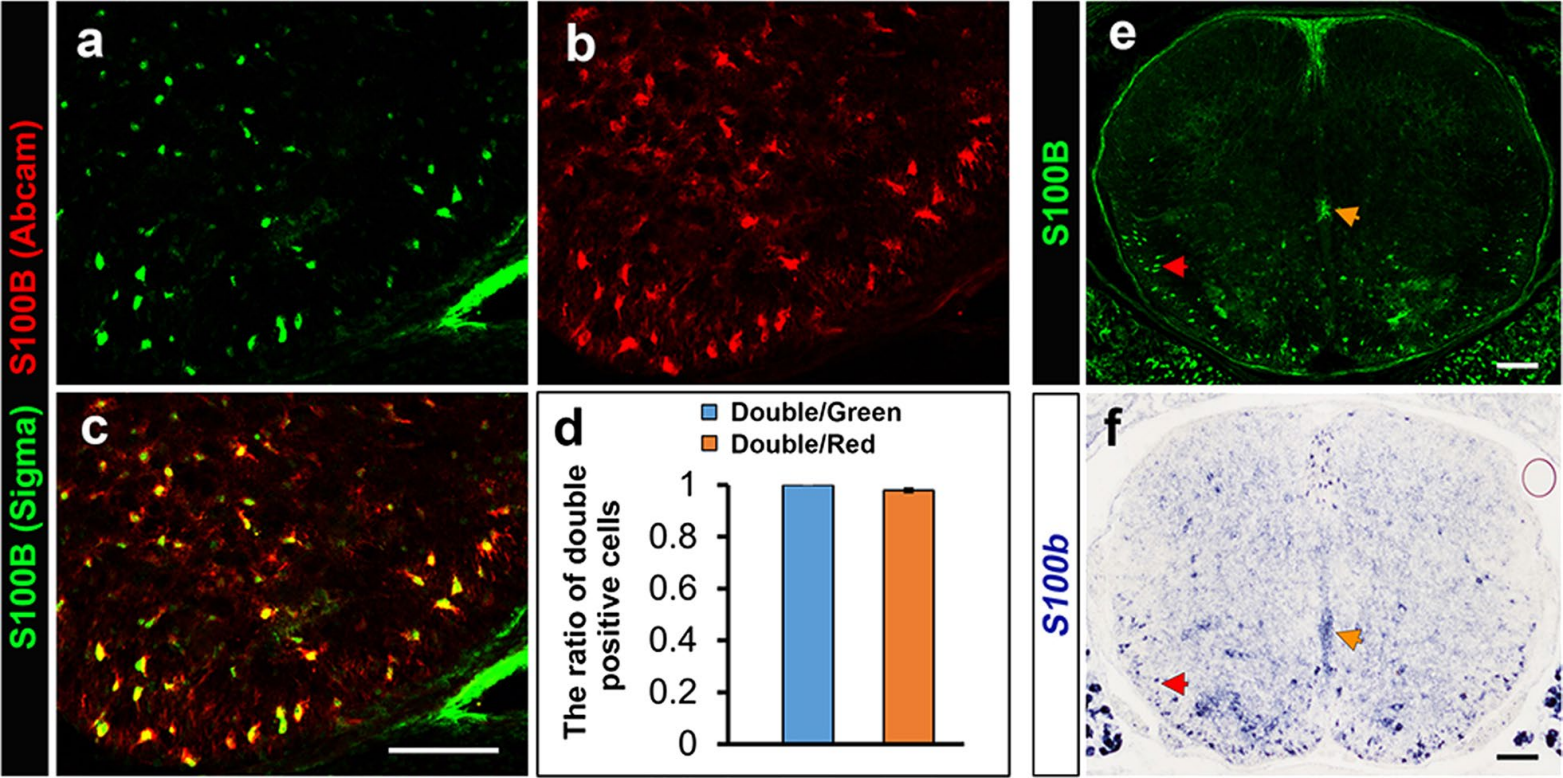
The specificity of S100B antibodies. **a**–**d** Sections of P0 mouse spinal cord were subject to anti-S100B immunostaining. The mouse-anti-S100B monoclonal antibodies (green) and rabbit-anti-S100B polyclonal antibodies (red) marked the same population of cells. **e**,** f** In situ hybridization against *S100b* mRNA (**f**) displayed a nearly identical expression pattern to that of immunostaining (**e**). Bar, 100 μm

Due to the wide use of S100B as an astrocyte marker and the conflicting reports of S100B expression in other glial cell types [24–27], it is necessary and important to investigate the specificity of S100B antibodies in details. Double immunolabeling on P0 mouse spinal cord sections with rabbit anti-S100B polyclonal antibodies (from Abcam) and mouse anti-S100B monoclonal antibodies (from Sigma) revealed that these two antibodies marked the same population of cells. Quantitatively, all S100B+ cells recognized by Sigma antibodies were labeled by Abcam S100B antibodies, and the ratio is 97.9 ± 0.8% vice versa, demonstrating their specific recognition of the same targe protein (Fig. 1a–d). Further, in situ RNA hybridization against *S100b* mRNA showed a nearly identical expression pattern to that of immunostaining (Fig. 1e, f), validating the use of these two antibodies for detection of S100B expression in the developing CNS tissues.

**Fig. 2:**
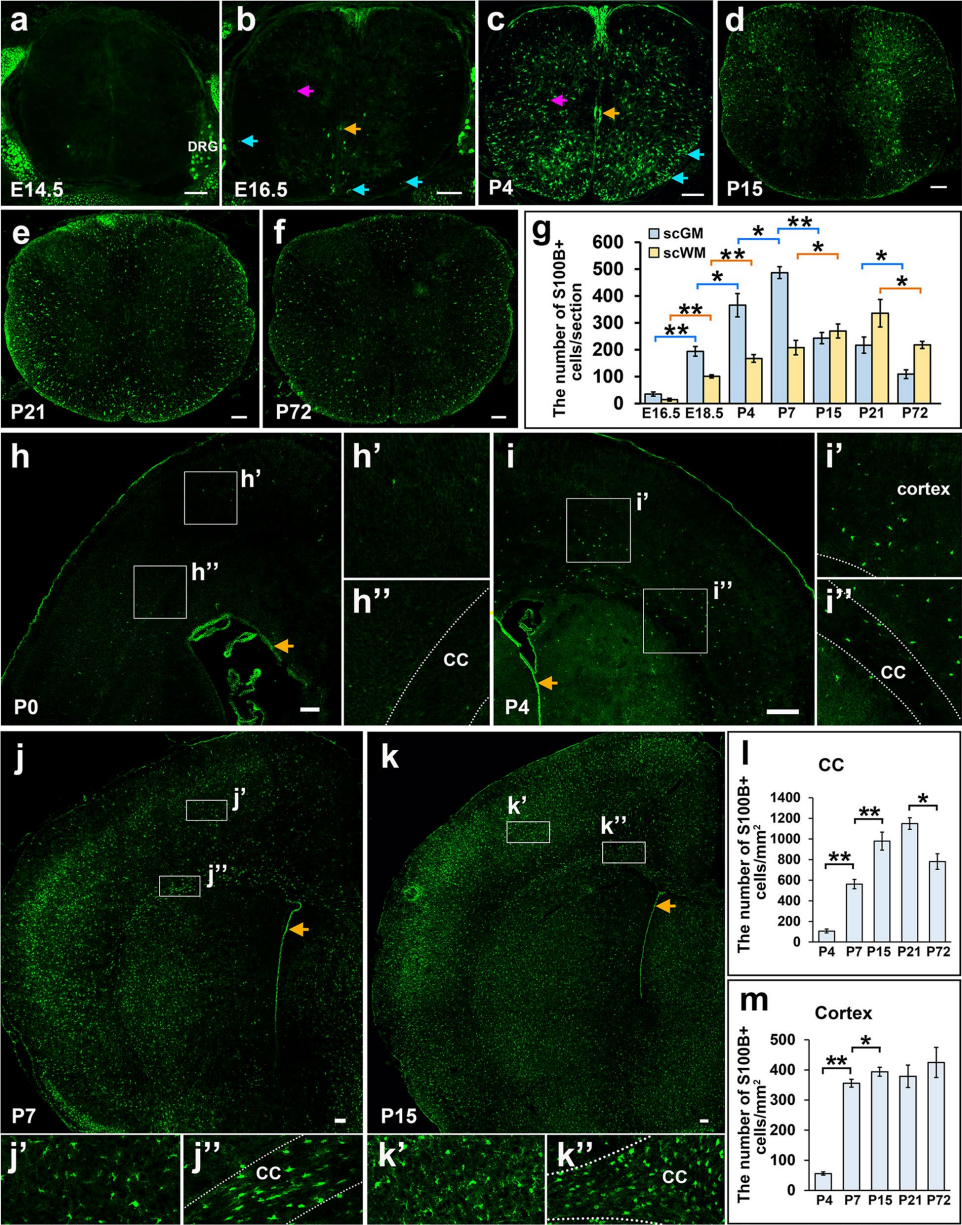
The expression pattern of S100B in the developing CNS. **a**–**f** Sections of E14.5, E16.5, P4, P15, P21 and P72 (adult) mouse spinal cords were subject to anti-S100B immunostaining. S100B+ ventricular cells are indicated with orange arrows (**b**, **c**). A few of S100B+ cells were detected both in the white matter (blue arrows) and gray matter (pink arrows) at E16.5 stage (**b**), but the number increased rapidly at postnatal stages until to P21. **g** Quantitative comparison of S100B expression in the gray and white matter of spinal cord at different developmental stages. **h**–**k** The expression of S100B in P0, P4, P7 and P15 forebrain. Orange arrows indicate the ventricular cells. Higher magnification images in the cortex and corpus callosum were shown in (**h′**–**k′**) and (**h″**–**k″**), respectively. **l**,** m** Quantitative comparison of S100B expression in the corpus callosum and cortex area of forebrain at different developmental stages. *sc* spinal cord, *WM* white matter, *GM* gray matter, *DRG* dorsal root ganglion, *CC* corpus callosum. n = 3, *p < 0.05, **p < 0.01. Bar, 100 μm

To investigate the developmental expression of S100B in the CNS, we first examined its expression in the spinal cord at different embryonic and postnatal stages by immunostaining. Prior to E14.5, S100B expression was not detected in the spinal cord tissues (Fig. 2a). At E16.5, S100B is expressed in the ventricular zone (orange arrows, Fig. 2b), and in a few scattered cells in the ventrolateral regions (Fig. 2b). Notably, some cells with weak S100B expression also appeared in the white matter at this stage (blue arrows, Fig. 2b). After birth, the number of S100B+ cells increased rapidly in the spinal tissue. In the gray matter region, S100B+ cells spread dorsally and occupied the entire spinal cord by P4, and their number reaches the peak at around P7 stage and then decreased gradually thereafter (Fig. 2c–g). In the meantime, the number of S100B+ cells in the white matter region increased gradually from E16.5 to P21, but decreased in adult spinal cord (Fig. 2c–g). Together, these expression studies suggested that S100B is primarily expressed in glial cells in the developing spinal cord.

In the developing forebrain, S100B expression is also detected in the ventricular neuroepithelial cells (orange arrow, Fig. 2h–k). In the brain parenchyma, sporadic cells with a low level of S100B expression could be observed in the cortical region at P0 stage (Fig. 2h–h″). Bright S100B+ cells emerged at about P4 stage both in the corpus callosum and cortical plate throughout the entire brain tissues (Fig. 2i–i″). By P7 stage, a large number of S100B+ cells were detected in most of the brain tissues, and their density reaches its maximum at P15 stage (Fig. 2j–j″, k–k″). Given that glial progenitor cells are predominantly produced before P0 stage in the cortical region, cortical astrocytes appear to acquire S100B expression during early postnatal stages. From P15 to adulthood, S100B+ cell density remains stable in the cortex (Fig. 2m), but gradually decreases in the corpus callosum regions (Fig. 2l), indicating that some of the white matter cells down-regulate S100B expression during development.

### S100B is not expressed in neurons

**Fig. 3:**
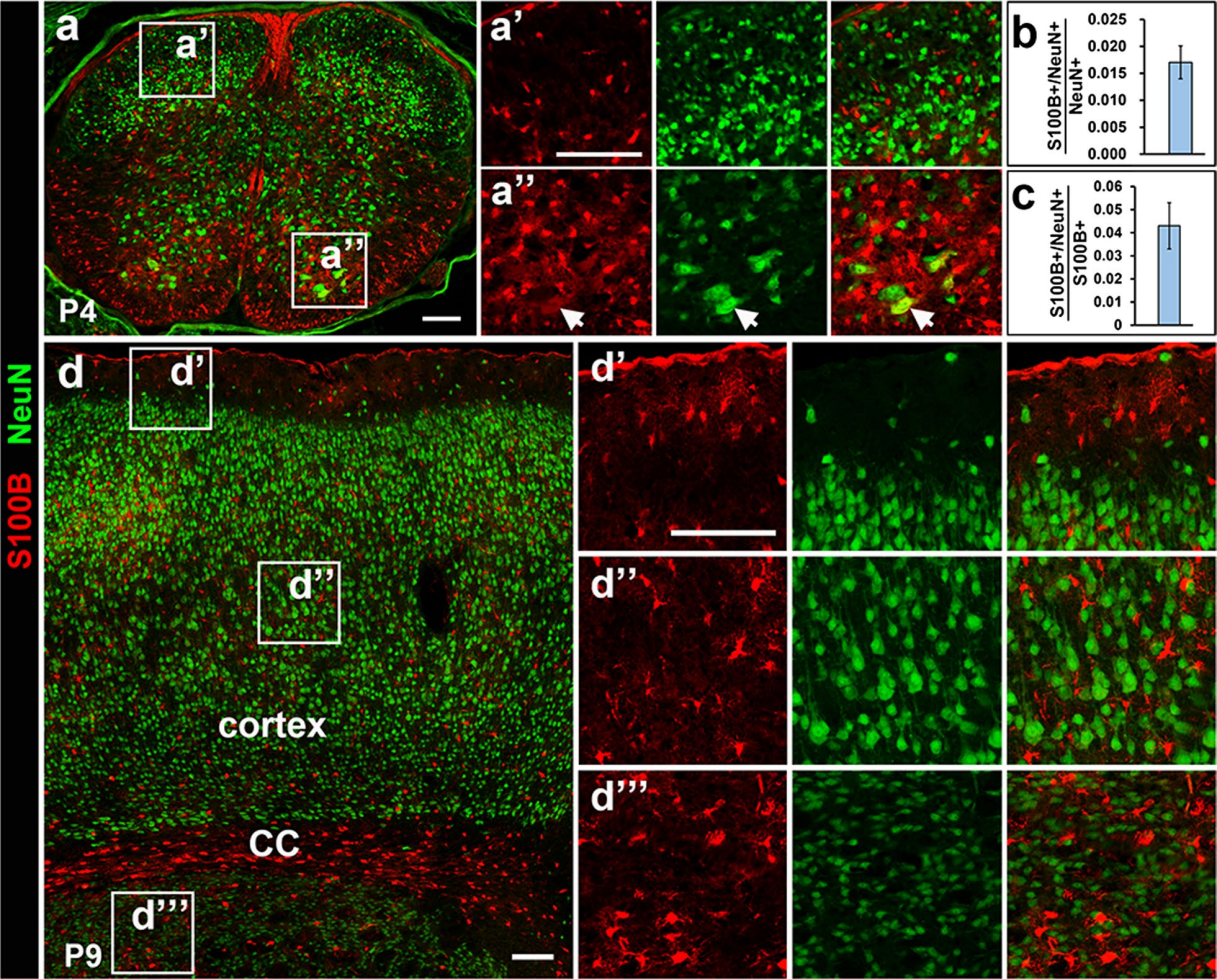
S100B is not expressed by neurons. **a**–**c** Spinal cord tissues from P4 stage were immunostained simultaneously with S100B and NeuN. Quantitative analysis was showed in (**b**,** c**). **d**–**d‴** None of neurons express S100B in different areas of brain tissues. *CC* corpus callosum. Bar, 100 μm

To examine whether S100B is expressed by postmitotic neurons, we performed anti-S100B and anti-NeuN double immunostaining in P4 spinal cord and P9 brain tissues, when S100B+ cells are distributed throughout the entire CNS tissues. Consistent with the previous reports that S100B is primarily expressed by glial cells [5, 24, 26], the vast majority of S100B+ cells do not co-express neuronal marker NeuN in the spinal cord tissue (Fig. 3a–a″), although a few large motor neurons in the ventral horn region are immunoreactive to S100B (1.7 ± 0.3% of gray matter S100B+ cells, and 4.3 ± 1.0% of total neurons) (arrows, Fig. 3a″, b, c). In the forebrain, none of S100B+ cells in the cortex and striatum regions were found to be NeuN+ neurons (Fig. 3d–d‴). Collectively, these double labeling experiments confirmed that S100B is exclusively expressed in non-neuronal cells except for a few spinal motor neurons.

### S100B was selectively expressed in protoplasmic astrocytes in the gray matter

**Fig. 4:**
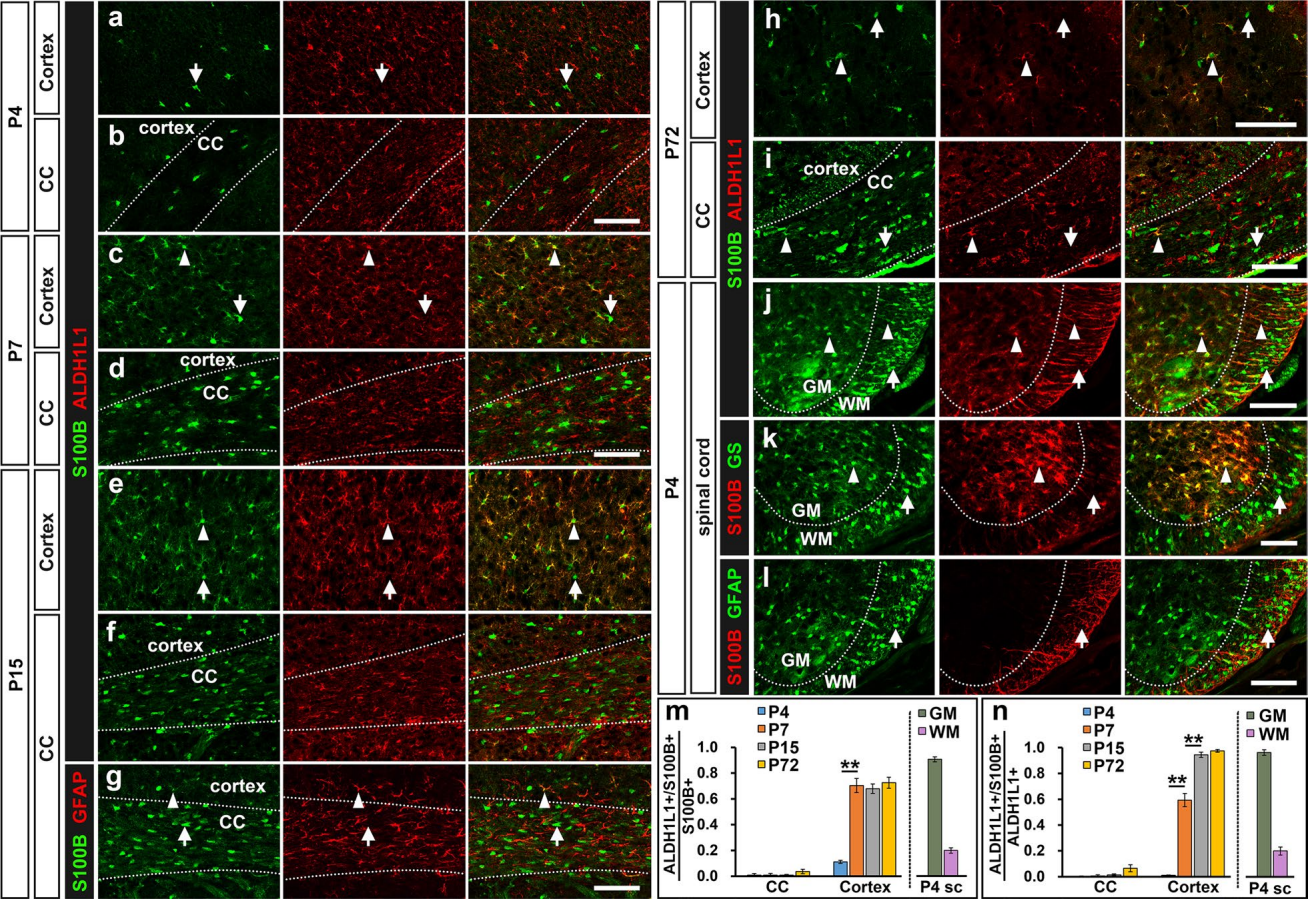
S100B is expressed in gray matter astrocytes but not white matter GFAP+ astrocytes. **a**–**i** Double labeling of S100B with ALDH1L1 and GFAP in the cortical region (**a**,** c**,** e**,** h**) and corpus callosum (**b**,** d**,** f**,** g**,** i**) of forebrain from P4 to adulthood. **j**–**l** Double labeling of S100B with ALDH1L1, GS and GFAP in P4 spinal cord. The arrow heads indicate the S100B+ astrocytes, and S100B+ cells that did not express astrocyte marker are indicated by white arrows. **m**,** n** Quantitative analysis of S100B expression in astrocytes. *sc* spinal cord, *WM* white matter, *GM* gray matter, *CC* corpus callosum. n = 3, *p < 0.05, **p < 0.01. Bar, 100 μm

To substantiate the expression of S100B in cells of astrocyte lineage, we performed double immunostaining with the ALDH1L1 antibody. ALDH1L1 is expressed by both astrocytes and their radial glial progenitors, and has been widely used as a specific pan-astrocyte marker in the parenchyma region outside the germinal zone [4, 28, 29]. In the spinal cord, we found that the majority of the S100B+ cells (90.5 ± 1.9%) in the gray matter co-expressed ALDH1L1 at P4 stage. Conversely, 96.4 ± 2.2% ALDH1L1+ cells in the gray matter were S100B+ (Fig. 4j, m, n). These findings confirmed the astrocyte identity of S100B+ cells in the spinal gray matter. Consistently, the vast majority of S100B+ cells in the gray matter were co-labeled with another astrocyte marker GS [30] (Fig. 4k). However, in the white matter region, only a small percentage of S100B+ cells (20.0 ± 3.1%) with low immunofluorescent intensity were co-stained with ALDH1L1 (arrow heads, Fig. 4j, m). Unexpectedly, the cells with a high level of S100B expression did not express ALDH1L1, GS or GFAP (arrows, Fig. 4j–l), suggesting that they are not astrocytes.

We next investigated the expression of S100B in the developing brain tissue. In the cortical region, S100B+ cells started to emerge at P4. At this stage, 1.1 ± 0.2% ALDH1L1+ astrocytes co-expressed S100B, and 11.1 ± 1.2% S100B+ cells express ALDH1L1 (Fig. 4a, m, n). However, at P7 stage, 70.4 ± 5.5% S100B+ cells were ALDH1L1+ in cortical region, and 59.3 ± 5.1% ALDH1L1+ cells were labeled by S100B (Fig. 4d, m, n), indicating a strong upregulation of S100B in astrocytes from P4 to P7 stage. By P15 stage, many S100B+ cells (67.9 ± 3.7%) in the cortical parenchyma co-stained with ALDH1L1 (arrow heads, Fig. 4e); conversely, the vast majority of ALDH1L1+ cells (94.4 ± 2.0%) are immunoreactive to S100B (Fig. 4e, m, n). In adult tissues, 72.5 ± 4.3% S100B+ cells were astrocytes, and 97.5 ± 1.0% ALDH1L1+ astrocytes were S100B+. Therefore, the majority of cortical astrocytes in the gray matter are S100B-expressing cells after P7 stage, and vice versa. S100B+ cells were also observed in the white matter (corpus callosum) of the cortex from P4 to adult stage. However, nearly all S100B+ cells in the corpus callosum did not express ALDH1L1 and GFAP in all periods that have been tested, and vice versa, refuting their astrocyte identity (Fig. 4b, d, f, g, i, m, n).

Taken together, these results demonstrated that S100B is mainly expressed by the protoplasmic astrocytes in the gray matter of CNS, and only a very small percentage of S100B+ cells represent astrocytes in the white matter region.

### S100 is specifically expressed in myelinating oligodendrocytes

**Fig. 5:**
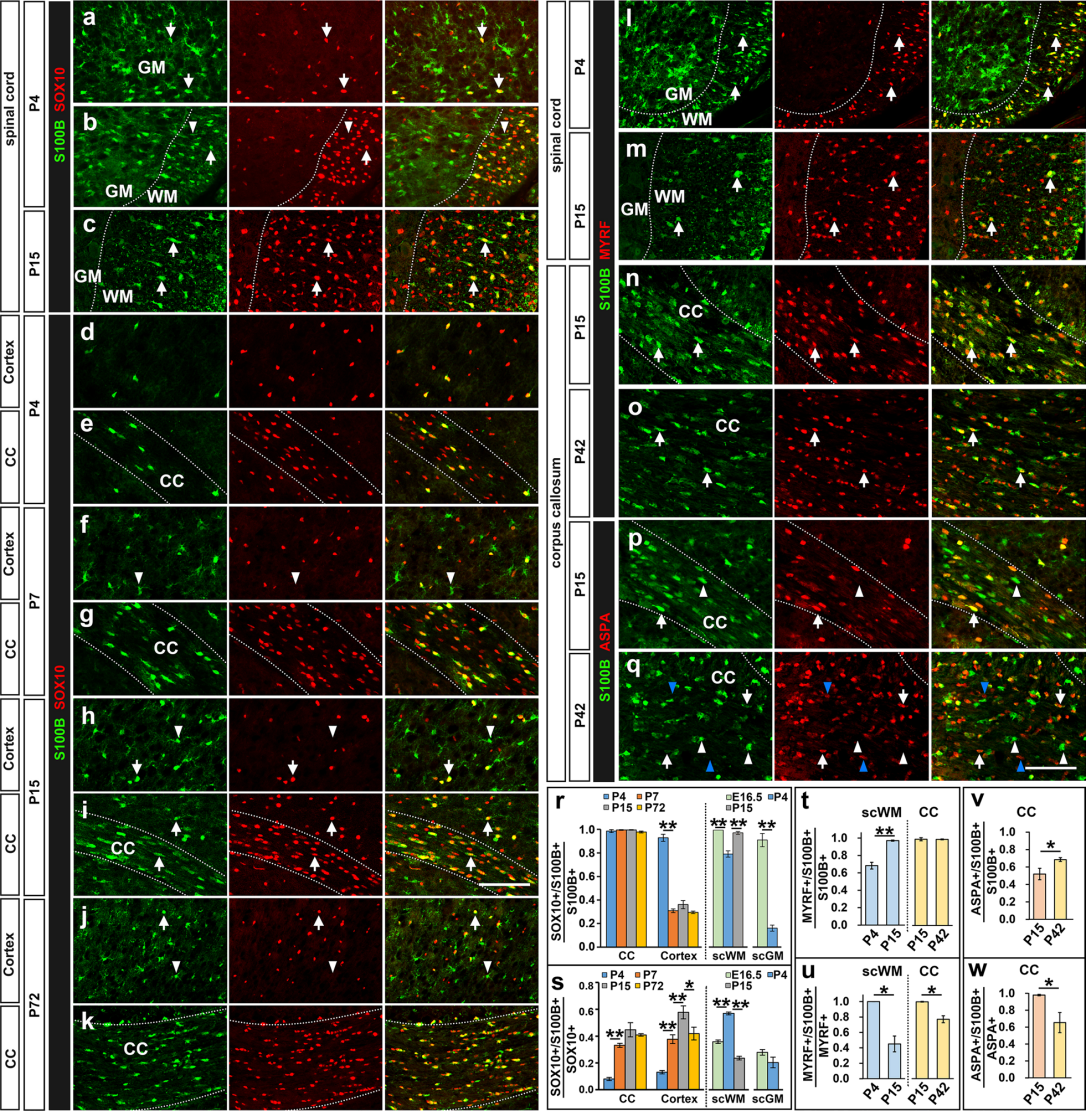
S100B is expressed in the differentiating oligodendrocytes. **a**–**k** Co-expression of S100B and SOX10 in mouse CNS tissues. Most S100B+ cells in spinal white matter and a few in the gray matter co-express SOX10 (arrows) (**a**–**c**). Similarly, nearly all the S100B+ cells are SOX10+ in the corpus callosum, and SOX10+/S100B+ cells are also presented in the cortex region (arrows) (**d**–**k**). White arrow heads refer to SOX10–/S100B+ astrocytes. **l**–**o** Co-expression of S100B and MYRF in mouse CNS tissues. Nearly all MYRF+ cells express S100B in P4 spinal white matter and P15 corpus callosum (arrows). **p**, **q** Co-expression of S100B and ASPA in brain corpus callosum. ASPA+ mature oligodendrocytes that have completely lost S100B expression are represented by blue arrow heads and ASPA–/S100B+ myelinating oligodendrocytes are indicated by white arrow heads. **r**–**w** Quantitative analyses of the co-expression of S100B with SOX10, MYRF and ASPA. *sc* spinal cord, *WM* white matter, *GM* gray matter, *CC* corpus callosum. n = 3, *p < 0.05, **p < 0.01. Bar, 100 μm

The lack of S100B co-expression in most ALDH1L1+/GFAP+ astrocytes in the white matter regions strongly suggest that S100B is expressed in cells of oligodendrocyte lineage. To investigate this possibility, we performed S100B double immunostaining with SOX10, a specific marker of oligodendrocyte-lineage cells [31], in P4 and P15 spinal cords. Double labeling experiments revealed a few SOX10+/S100B+ cells in the gray matter of spinal cord (arrows, Fig. 5a). At P4 stage, about 80 % white matter S100B+ cells (79.6 ± 2.7%) were co-labeled with SOX10; conversely, around 60% white matter SOX10+ cells (59.0 ± 1.1%) were positive for S100B (Fig. 5b, r, s). Meanwhile, nearly all these double positive cells displayed a high level of S100B expression. By P15 stage, nearly all the S100B+ cells (97.6 ± 1.2%) in the white matter were co-labeled with SOX10 (Fig. 5c, r). Thus, the vast majority of S100B+ cells in the white matter represent oligodendroglial cells, while only a tiny fraction of astrocytes displays a weak and transient expression of S100B.

As oligodendroglial differentiation in the forebrain generally occurs later than that in the spinal cord, we carried a similar double labeling experiment in cortical tissues from P4 to adult stage. At P4 stage, nearly all S100B+ cells (98.6 ± 1.3% in corpus callosum and 92.9 ± 3.0% in cortical plate) co-expressed SOX10 (Fig. 5d–e, r). From P7 to P15, more than 99 % S100B+ cells in the corpus callosum are labeled with SOX10, but the ratio in the cortical plate decreased rapidly to about 30% due to its rapid upregulation in cortical astrocytes as described above (Fig. 5f–i, r). In adulthood (P72), the ratio was unchanged in both the cortical gray matter and corpus callosum region (Fig. 5j, k, r). Based on these detailed expression studies, we conclude that S100B is also expressed in oligodendrocyte-lineage cells, especially in the white matter region of CNS.

The gradual upregulation of S100B expression in SOX10+ cells suggest its selective expression in differentiating and/or mature oligodendrocytes. This possibility was investigated by its co-labeling with MYRF, a transcription factor that is specifically expressed by oligodendrocytes at the onset of their differentiation [32]. As expected, all MYRF+ cells are S100B+ in the white matter of P4 spinal cord and in P15 corpus callosum (Fig. 5l, n, u). However, the percentage of MYRF+ cells that co-expressed S100B in white matter was lowered in P15 spinal cord (Fig. 5m, u) and in P42 corpus callosum, and nearly all S100B+ cells express MYRF at these stages (Fig. 5m, o, t). Together, these results suggest S100B is specifically up-regulated in MYRF+ newly differentiated oligodendrocytes, but gradually downregulated during subsequent maturation process. Consistent with this hypothesis, nearly all ASPA+ mature oligodendrocytes [33] co-expressed S100B in P15 corpus callosum (Fig. 5p, w), but only about two third of them still expressed S100B (arrows, Fig. 5q) and many ASPA+ cells did not express S100B in the corpus callosum at P42 stage (blue arrow heads, Fig. 5q, w). In addition, it was noted that the ASPA–/S100B+ cells expressed a higher level of S100B than ASPA+/S100B+ cells at P42 stage (white arrow heads, Fig. 5q), providing further support for down-regulation of S100B in the ASPA+ mature oligodendrocytes.

**Fig. 6:**
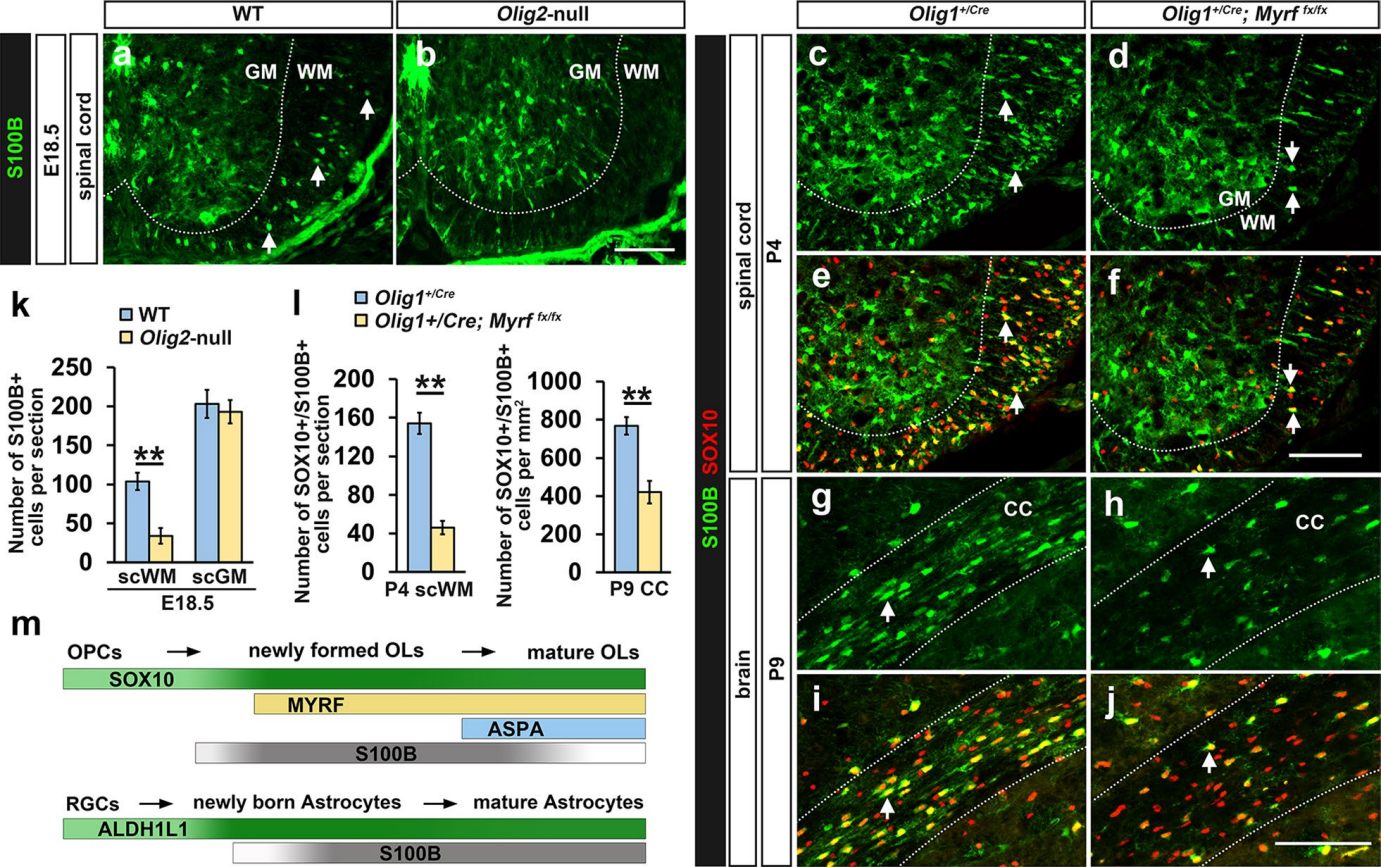
S100B expression in *Olig2*-null and *Myrf* conditional knockout mutants. **a**,** b** S100B+ cells were dramatically reduced in E18.5 *Olig2*-null spinal cord in the white matter, but not in the gray matter. **c**–**j** The number of S100B+/SOX10+ oligodendrocytes was significantly reduced in the white matter of spinal cord and forebrain (corpus callosum) in *Myrf* conditional knockout mutants. **k**–**l** Statistical analysis of the number of S100B+ oligodendrocytes in *Olig2* and *Myrf* mutants. SOX10+/S100B+ cells are represented by white arrows. **m** Schematic diagram of the time window of S100B expression in differentiating oligodendrocytes and gray matter astrocytes. In oligodendrocyte lineage, S100B expression is slightly earlier than that of MYRF, and gradually down-regulated in ASPA+ mature oligodendrocytes. In astrocyte lineage, S100B is gradually up-regulated and maintained in adulthood. *sc* spinal cord, *WM* white matter, *GM* gray matter, *CC* corpus callosum, *OLs* oligodendrocytes, *OPCs* oligodendrocyte progenitor cells, *RGCs* radial glial cells. n = 3, *p < 0.05, **p < 0.01. Bar, 100 μm

The specific expression of S100B in oligodendrocytes in the white matter was also corroborated in *Olig2*-null and *Myrf* conditional knockout mutants. It was previously reported that the generation of oligodendrocyte progenitor cells (OPCs) are completely prevented in *Olig2* mutant mice, leading to an absolute loss of oligodendrocyte population in the spinal cord [34–36]. Consistent with this, the number of S100B+ cells were dramatically reduced in the white matter of *Olig2*-null spinal cord, but not in the gray matter (Fig. 6a, b, k). In addition, it was previously shown that oligodendrocyte development stalls at premyelinating stage in the absence of *Myrf* expression [32]. In keeping with the notion that S100B is expressed in differentiating oligodendrocytes, expression of S100B was dramatically decreased in the white matter of *Olig1*^*+/Cre*^; *Myrf*^*fx/fx*^ mutant tissues (Fig. 6c–j, l). These genetic studies confirmed the selective expression of S100B in cells of oligodendrocyte lineage in the white matter.

## Discussion

S100B has been widely used as a molecular marker specific for astrocytes in both developing and adult CNS tissues. In this study, we presented the compelling molecular evidences that S100B is expressed in cells of both astrocyte and oligodendrocyte lineages at various developmental stages, thus provided the detailed information about the specificity and efficiency of S100B as an astrocyte marker in both spinal cord and brain tissues. In the spinal cord, S100B is predominantly expressed in protoplasmic astrocytes in the gray matter. By contrast, the vast majority of S100B+ cells in the white matter of postnatal spinal cord are SOX10+/MYRF+ myelinating oligodendrocytes. Similarly, most of S100B+ cells in the cortical plate are ALDH1L1+ astrocytes. Surprisingly, S100B is not expressed in the GFAP+ fibrous astrocytes at all in the corpus callosum, contrary to the previous belief (Fig. 4g). Instead, S100B is selectively expressed in SOX10+ differentiating OPCs and newly differentiated oligodendrocytes. Interestingly, a fraction of the S100B+ cells (~ 30%) in the gray matter are also labeled by SOX10 (Fig. 5g, i, k, r), especially in the area adjacent to corpus callosum or in the regions enriched with differentiating oligodendrocytes, demonstrating its expression in gray matter differentiating oligodendrocytes as well. In general, S100B+ cells are composed of myelinating oligodendrocytes and gray matter protoplasmic astrocytes in the CNS.

Furthermore, only a portion of SOX10+ oligodendroglial cells are S100B+ in different CNS regions and at different developmental stages (Fig. 5s), proving that S100B is not abundantly expressed by immature OPCs, but upregulated in cells of oligodendrocyte lineage during differentiation stage. It is worth noticing that nearly all S100B+ cells are labelled with SOX10 in E16.5 spinal cord and P4 brain tissues both in the gray and white matter regions (Fig. 5l), indicating that S100B expression in oligodendrocyte lineages occurs earlier than that in astrocytes, even in the gray matter. Also, the observation that all cells with low level of S100B expression are SOX10+ in spinal white matter at E16.5, a stage prior to MYRF expression (blue arrows, Fig. 2b), which was also observed in P0 forebrain (Fig. 2h), suggests that the expression of S100B in pre-differentiating OPCs (Fig. 6m). Consistent with this idea, many SOX10+ oligodendrocytes still maintain S100B expression in *Myrf* conditional mutants (Fig. 6c–j). Further, we showed that S100B is co-expressed with ASPA for a brief period of time, followed by a gradual down-regulation in myelinating and myelinated oligodendrocytes (Figs. 5p, q, w and 6 m).

Consistent with the earlier findings, S100B primarily labels ALDH1L1+/GS+ protoplasmic astrocytes in the gray matter (Fig. 4). Notably, strong expression of S100B in gray matter astrocytes can only be detected after E16.5 in the spinal cord and P4 in the forebrain (Fig. 2) when newborn astrocytes are widely distributed in these tissues, suggesting a lagging expression of S100B in protoplasmic astrocytes (Fig. 6m). Considering that S100B is predominantly localized in the cell body and suitable for cell counting, it could still be used as an alternative marker for gray matter astrocytes when combined with SOX10 to exclude its labeling of S100B+ oligodendrocytes.

Previous studies also reported the expression of S100B in neurons [24, 27] and OPCs [25]. However, we failed to identify S100B+/NeuN+ neurons in mouse cortical region (Fig. 3). One plausible explanation for this discrepancy could be the higher sensitivity caused by S100B promoter sequence used in S100B-EGFP transgenic mice, as noticed in these studies [24, 27]. Likewise, our data did not support a broad expression of S100B in immature OPCs, since only a part of SOX10+ oligodendroglial cells are immunoreactive to S100B. However, our results still implied a low level of S100B expression in pre-differentiating OPCs as mentioned above.

Our studies clearly demonstrated that S100B labels two distinct types of glial cells in the gray matter and white matter, and therefore can not be used as a universal marker for astrocytes. The function of S100B in neural development and functioning remains largely unknown at this stage. As a result, the functional significance of the shared expression of S100B in gray matter astrocytes and differentiating oligodendrocytes is not clear. It may reflect their common developmental path as both cell types become highly branched during morphogenesis, or reflect their similar physiological functions such as providing metabolic supports for neurons. In analogy, the differential expression of S100B in gray and white matter astrocytes in the CNS may reflect their different morphogenetic process or their distinct functions in the CNS, giving that the S100B+ protoplasmic astrocytes are highly branched and intimately associated with neuronal cell bodies while the GFAP+/S100B– fibrous astrocytes are less branched and in close contacts with myelinated axons. Consistent with this idea, it is reported that S100B regulates astrocyte morphology in vitro depending on the intracellular regulatory activities of this protein [37].

In the peripheral nervous system (PNS), S100B is also expressed in the satellite cells and Schwann cells [38, 39]. Therefore, S100B is enriched in several distinct types of glial cells in both CNS and PNS. Since S100B is a secreted Ca^2+^-binding protein and has neurotrophic activity in enhancing neurite outgrowth and neuronal survival [20, 40, 41], it may function as an secreted extracellular signal influencing the development and function of neurons and this possibility awaits to be investigated in future genetic studies.

## Conclusions

This study presents several lines of molecular and genetic evidences that S100B is selectively expressed in both protoplasmic astrocytes and myelinating oligodendrocytes in the developing CNS. Our detailed expression analyses revealed that S100B+ cells in the gray matter are composed of SOX10+ myelinating oligodendrocytes and ALDH1L1+ protoplasmic astrocytes. In the white matter regions, nearly all the S100B+ cells are SOX10+/MYRF+ myelinating oligodendrocytes in the developing CNS. Based on these findings, we conclude that S100B is not a specific marker for astrocyte lineage; instead, it is an alternative marker for gray matter protoplasmic astrocytes when SOX10 expression is excluded.

## Methods

### Immunofluorescence and in situ hybridization

Tissues were fix in 4% PFA overnight at 4 ℃, and transferred into 20% sucrose in PBS overnight at 4 ℃. Tissues were then embedded in optimum cutting temperature compound (OCT) (ThermoFisher, #6506) and sectioned into 14 μm on a cryostat. Experimental procedures for Immunofluorescence and in situ hybridization were described previously [42]. To produce *S100b* riboprobe, 1009 bp fragments corresponding to 262–1270 nt of mouse *S100b* mRNA (NCBI Reference Sequence: NM_009115) were cloned into pBlueScript KS(–) vectors for transcription in vitro.

For immunofluorescence, tissue sections of P15, P21, P42, P72 mice were first washed in PBS for 10 min and incubated in citrate antigen retrieval solution (Sangon Biotech, E673001) at 85 ℃ for 30 min and then cool down to room temperature. This heat-induced peptide retrial step is not required for the sections from young animals. Thereafter, slides were blocked in 5% goat serum at room temperature for 1 h, and incubated with primary antibodies (diluted in 5% goat serum in PBS, 0.1% Triton X-100) overnight at 4 ℃. On the following day, sections were washed in PBS for 3 times, incubated with secondary antibodies for 1 h at room temperature (diluted in 5% goat serum in PBS, 1:2000), and washed in PBS for 3 times for a total 15 min before being mounted. Antibodies were used as follows: Rabbit-anti-S100B (Abcam, ab15520, 1:500, for double immunostaining), Mouse-anti-S100B (Sigma, S2657, 1:500), anti-GS (Chemicon, MAB302, 1:500), mouse-anti-NeuN (Millpore, MAB377, 1:500), Rat-anti-MYRF (Oasis Biofarm, OB-RT003), Rat-anti-ALDH1L1 (Oasis Biofarm, OB-RT002), Guinea pig-anti-SOX10 (Oasis Biofarm, OB-GP001), Rat-anti-ASPA (Oasis Biofarm, OB-RT005). The Alexa Fluor 488 and 594 secondary antibodies were purchased from Thermo Scientific (A21125, A21121, A11012, A11034, A11007, A11006, A11076, A11073).

### Mice

Use of the animals was approved by the Committee of Laboratory Animals, Hangzhou Normal University. Mouse lines for *Olig2*-null, *Olig1-Cre* and *Myrf*^*flox*^ were described previously, as well as the genotyping methods [32, 34, 35].

### Statistical analysis

Three mice were used in each experiment. All the cells labeled by specific markers on the spinal cord sections were counted. In the brain tissues, cells were calculated in the regions described in the text, and the results were presented as the cell density (cell number per mm^2^). Data were presented as mean ± standard deviation (SD). The two-tailed Student’s t-test was performed to determine whether differences in cell numbers were statistically significant. Error bars represented the standard deviations. Statistical significance was considered to be at *p < 0.05, **p < 0.01, n = 3.

## Data Availability

All date generated during this study are included in this article.

## Abbreviations

S100B: S100 protein, beta polypeptide, neural
SOX10: SRY (sex determining region Y)-box 10
ALDH1L1: Aldehyde dehydrogenase 1 family, member L1
GFAP: Glial fibrillary acidic protein
MYRF: Myelin regulatory factor
ASPA: Aspartoacylase
GS: Glutamine synthetase
Olig1/2: Oligodendrocyte transcription factor 1/2
CNPase: 2′3′-Cyclic nucleotide-3′-phosphohydrolase
NG2: Chondroitin sulfate proteoglycan 4
EGFP: Enhanced green fluorescent protein
CNS: Central nervous system
PNS: Peripheral nervous system
OPCs: Oligodendrocyte progenitor cells
sc: Spinal cord
WM: White matter
GM: Gray matter
CC: Corpus callosum
SD: Standard deviation
